# Emerging Paradigms in Fetal Heart Rate Monitoring: Evaluating the Efficacy and Application of Innovative Textile-Based Wearables

**DOI:** 10.3390/s24186066

**Published:** 2024-09-19

**Authors:** Md Raju Ahmed, Samantha Newby, Prasad Potluri, Wajira Mirihanage, Anura Fernando

**Affiliations:** Department of Materials, The University of Manchester, Manchester M13 9PL, UK; mdraju.ahmed@manchester.ac.uk (M.R.A.); samantha.newby@postgrad.manchester.ac.uk (S.N.); prasad.potluri@manchester.ac.uk (P.P.); wajira.mirihanage@manchester.ac.uk (W.M.)

**Keywords:** fetal, heart rate, congenital disabilities, textile-based, wearable, early detection

## Abstract

This comprehensive review offers a thorough examination of fetal heart rate (fHR) monitoring methods, which are an essential component of prenatal care for assessing fetal health and identifying possible problems early on. It examines the clinical uses, accuracy, and limitations of both modern and traditional monitoring techniques, such as electrocardiography (ECG), ballistocardiography (BCG), phonocardiography (PCG), and cardiotocography (CTG), in a variety of obstetric scenarios. A particular focus is on the most recent developments in textile-based wearables for fHR monitoring. These innovative devices mark a substantial advancement in the field and are noteworthy for their continuous data collection capability and ergonomic design. The review delves into the obstacles that arise when incorporating these wearables into clinical practice. These challenges include problems with signal quality, user compliance, and data interpretation. Additionally, it looks at how these technologies could improve fetal health surveillance by providing expectant mothers with more individualized and non-intrusive options, which could change the prenatal monitoring landscape.

## 1. Introduction

Defects in the heart are the most common congenital disease that results in death. Statistics show that nearly 1% of babies have congenital heart defects in any form [1]. These defects are considered simple but ultimately affect long-term health; for instance, defects may become severe after several years and result in death. The generation of congenital heart defects starts at a very early stage of pregnancy, where the formation of the heart can cause damage to any body part and even damage the functions of the heart [2,3,4]. Several reasons behind these cardiac anomalies include inherited disorders, genetic syndromes, and environmental factors, including misuse of drugs and several other infections [5]. As a result, fetal heart monitoring has become an effective tool for detecting early-stage defects such as ischemia [6]. Medical professionals have long recognized the difficulties in monitoring fetal well-being because the position of the fetus within the uterus, surrounded by the amnion and amniotic fluid, renders direct inspection of the fetus highly challenging for most diagnostic procedures [7,8].

**Figure 1:**
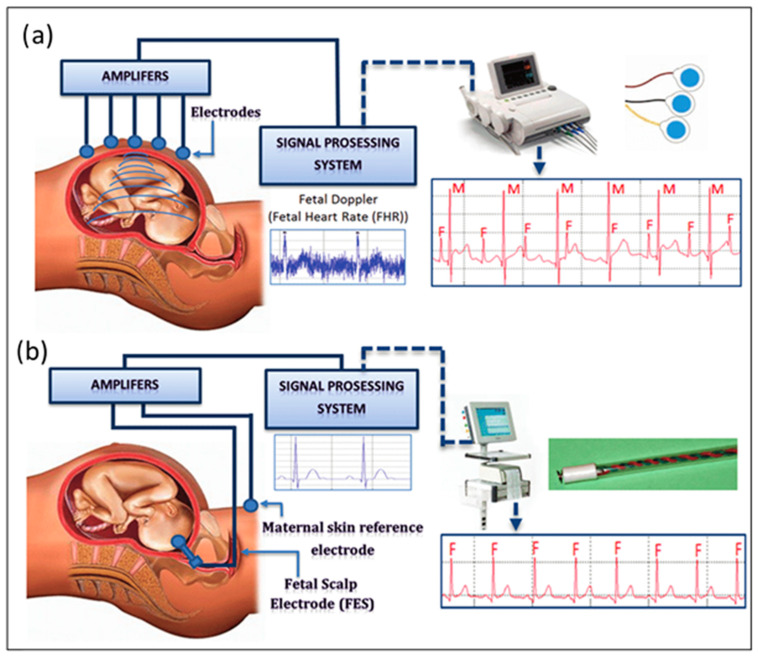
(**a**) Non-invasive fHR measurement. (**b**) Invasive fHR measurement, reproduced from [19] under terms of the Creative Commons Attribution License.

To monitor fetal heart rate (fHR), there are two determined methods, invasive and noninvasive, as seen in Figure 1. Among them, noninvasive monitoring gained ultimate popularity due to indirect contact with the baby’s body through methods such as fetal ultrasounds and maternal electrocardiograms (ECGs) [9]. Although monitoring the fHR through these noninvasive approaches is safe, several challenges limit their advantages, like myographic signal, the brain activity of the fetus, several dielectric layers of bio and physical structures, and motion artefacts contaminating the heart signals [10]. Specific cardiac defects, such as arrhythmias, have particular manifestations in the morphology of electrical signals generated by the heart. This morphology of a cardiac electric signal, recorded by an ECG, contains more information than the other traditional sonographic methods [11].

Recently, wearable textile-based monitoring systems have gained attention as effective tools for the early detection of complications and interventions and for recording and evaluating the effectiveness of the interventions and therapies [12,13,14]. These textile-based sensors usually comprise flexible structures created through weaving, knitting, and embroidery technology, which may involve different sensor printing technologies [15]. Careful integration of sensors into garments can make fHR measuring and monitoring more practical, comfortable, reliable, and non-invasive. These sensors, which are manufactured for healthcare machines and dedicated to measuring the fetal body’s bio-potentials, are made from electrodes and are found in ECG, electromyography (EMG), electroencephalography (EEG), etc. [15].

Conventional fetal heart monitoring during pregnancy is usually performed under the supervision of medical practitioners; this is specialized and may have scarce direct manpower resources, which can result in difficulties identifying potential physiological fetal and maternal health conditions. Moreover, existing signal processing methods are unreliable in providing undistorted fetal electrocardiogram signals from the mother’s abdomen due to the low signal-to-noise ratio (SNR) recorded from the maternal body [16]. Additionally, current textile-based sensors often show poor washability, poor skin–electrode contact, and sensitivity to body movements, causing motion artefacts [17]. Traditionally used electrodes are made of electroconductive gels and are unsuitable for wearable textile devices because of the fast performance reduction over time due to high contamination and evaporation rates [18]. Over the previous decades, several researchers have tried to develop wearable electrodes with improved performance; however, few research teams have looked at creating textile-based sensors for fHR monitoring. Therefore, this paper will look into the techniques and applications required for fHR monitoring and the few patents obtained and research conducted for textile-based sensors.

## 2. Measuring Techniques of fHR

It is reported that fHR monitoring during pregnancy reduces fetal loss, perinatal morbidity, and maternal distress [20,21]. Several approaches can be used to analyze fHR, including a fetoscope, a Doppler ultrasound device, a cardiotocography (CTG), and a fetal ECG (fECG). In fECG, the electrodes can be attached in two ways, intrauterine and directly to the fetus’ scalp, which can only be used during birth, as the invasive procedure can harm the mother or fetus, or by attaching the electrodes to the mother’s abdomen in a less-invasive method [22]. It is necessary, however, to ensure that the mother’s signals are not misinterpreted as the fHR signals. Table 1 shows the variability between the two.

### 2.1. Fetal Electrocardiography (fECG)

**Figure 2:**
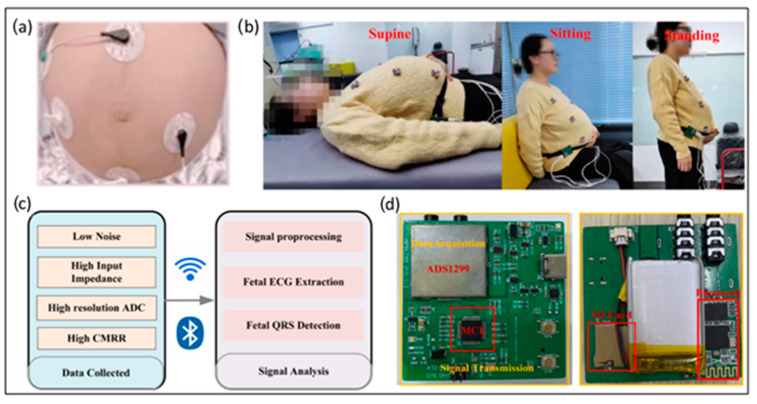
Wearable fetal ECG monitoring system from abdominal ECG recording. (**a**) Electrode placement. (**b**) Postures of the subject. (**c**) Monitoring system. (**d**) Hardware prototype. Reprinted from [30] under terms of the Creative Commons Attribution License.

One technique for recording the electrical activity of the fetus’s heart is fetal ECG (fECG), shown in Figure 2, which enables the evaluation of the fetus’s health during pregnancy and labor. Using ECG to find the fetus’s health was initially determined by using a Willem Einthoven-created string galvanometer, a device intended to record the electrical activity of adult hearts to measure the electric signal from the fetus’s heart [23]. Cremer’s research placed electrodes on the pregnant mother’s abdomen, vagina, esophagus, and rectum to obtain the fECG signal. In this manner, he demonstrated the diagnostic capabilities of fetal heart monitoring. Due to unwanted background interference, such as the maternal ECG waveform, noise from adjacent tissues, and the device, the quality of his recordings was poor, and, in practice, the low quality did not permit accurate fetal assessment. Over the last 40 years, many researchers have struggled with the problem of isolating the fetal fECG signal, although modern computers, amplifiers, and dedicated softwares help to obtain a more precise signal, a complete image of the atrioventricular complexes of the fetal heart, and typical fECG traces of the fetal cardiac cycle [24]. In 1953, Smyth et al. used an electrode attached to the amniotic membranes for the first time to detect fECG, while Hon et al. in 1962 designed an electrode that could be connected directly to the fetal scalp or other presenting parts of the fetus, making it possible to evaluate the characteristics of P (atrial depolarization) and T (ventricular repolarization) waves of a fECG more precisely [25].

Currently, fECG analysis is used in the clinical domain to analyze the heart rate and its associated variability. Despite the richness of the literature, several vital areas of fECG still require further study, particularly in the multichannel, non-invasive maternal abdominal measurements. Reliably recording fetal ECG (fECG) patterns for in-depth morphological study is the main objective. But a number of obstacles prevent this from happening.

- Low-amplitude fECG results are caused by weak cardiac impulses and poor conductivity of the fetal layers on the surface of the mother’s body [26].
- Maternal ECG, uterine contractions, respiratory activity, and motion artefacts cause interference [27].
- Challenges include fetal movements and the need for a consistent fetal cardiac representation along the body axis [28].
- It is crucial to develop automated processes that can be applied to large datasets with little expert assistance.
- Key parts include creating criteria for predicted cardiac signals’ degree of confidence and setting theoretical boundaries for the data that can be gleaned from body surface recordings despite noise [29].

### 2.2. Cardiotocography (CTG)

Fetal HR monitoring is complex for obstetricians to determine whether the fetus continues in a healthy state as the pregnancy progresses [20]. The standard approach in the clinical assessment of fetal well-being is cardiotocography (CTG), which quantifies fHR based on ultrasound pulses. In the clinical routine, the course of the fHR during data acquisition is qualitatively evaluated by visual inspection of the CTG track, categorization of base heart rate and variability, accelerations and delays, and sinusoidal patterns [31].

CTG is an accepted clinical practice for non-invasive tests for prenatal monitoring, even though it may only provide imprecise fHR signals [32]. The fHR signal is typically measured and identified manually, using morphological characteristics that are reviewed visually for quantitative and qualitative attributes. However, this method has limitations because it is subject to the physician’s knowledge and lacks impartiality and trustworthiness [33].

Furthermore, the CTG examination requires a hospital setting for execution, as it requires a skilled clinician to interpret the clinical findings and utilize specialized signal-recording technology. Nevertheless, CTG’s clinical advantages regarding outcomes such as perinatal mortality or the incidence of caesarean section remain unclear. CTG has certain limitations concerning quantifying short-term HRV, particularly concerning differences in the duration of consecutive heartbeats. The widely used CTG devices for monitoring fetal heart activity also use the ultrasound Doppler technique, in which an ultrasound beam is directed at the fetus, whose heart movement determines the reflection used to estimate fHR. However, these devices are unsuitable for long-term monitoring, as uncontrolled high-dose exposure can have harmful effects, and, when the baby moves, the probe will have to be repositioned.

Moreover, using ultrasonic Doppler in CTG devices raises questions regarding safety and practicality for continuous, long-term monitoring, even though it is effective for short-term assessments. The possible risks of prolonged ultrasound exposure, such as tissue heating and cavitation effects that could endanger fetal development, have been discussed [34,35]. These risks are especially relevant during the critical stages of pregnancy when continuous monitoring is frequently desired but tricky to accomplish with available CTG technology. The vulnerability of CTG to signal loss or degradation as a result of fetal movements or maternal factors, such as obesity, is another major disadvantage [36]. These factors can interfere with the ultrasound signal, causing inaccurate readings or necessitating frequent equipment adjustments. This might jeopardize the standard of care by interfering with the monitoring process and raising the possibility of false alarms or essential events being missed. Additionally, manual interpretation of CTG data is inherently subjective and heavily reliant on the clinician’s experience and expertise. This subjectivity can lead to variations in diagnoses and treatment decisions because different practitioners may interpret the same data differently. The lack of standardization in interpreting CTG results complicates its usefulness as a diagnostic tool, potentially leading to inconsistent clinical outcomes.

Despite these limitations, CTG remains a popular tool in prenatal care due to the lack of more reliable and non-invasive alternatives for continuous fetal monitoring. However, there is a clear need for more advanced, objective, and safe monitoring technologies, particularly those that can provide constant, long-term assessment without the risks associated with extended ultrasound exposure or the variability in manual data interpretation.

### 2.3. Phonocardiography (PCG)

**Figure 3:**
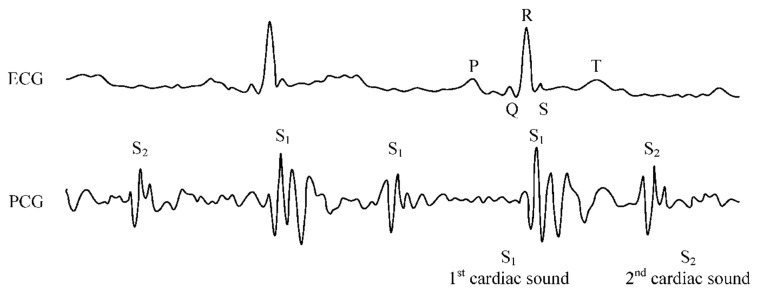
Sample recordings of ECG which includes the P wave, QRS complex, and T wave; these help clinicians assess the timing and magnitude of electrical impulses that coordinate the heartbeat and PCG signals where the first heart sound (S1) corresponds to the closure of the mitral and tricuspid valves at the start of systole, while the second heart sound (S2) corresponds to the closure of the aortic and pulmonary valves at the end of systole additional sounds, such as S3 and S4, which can indicate pathological conditions. Reprinted from [38] under terms of the Creative Commons Attribution License.

Kergardec, Marsac, and Kennedy discovered fetal phonocardiography (PCG) during the 17th century [37]. The generation of various characteristic sounds accompanies the heart’s mechanical activity. The mechanical activity of the heart is accompanied by the production of distinctive sounds that are related to variations in blood flow velocity and the opening and closing of heart valves. These acoustic waves, often known as heart sounds, are analyzed using PCG, a diagnostic method. Figure 3 shows that these noises are accompanied by mechanical vibrations in the heart’s arteries.

In a general sense, ECGs and PCGs serve as crucial diagnostic and monitoring tools to characterize cardiac electrical and mechanical behaviors, respectively. This categorization is linked to the methodology employed for measurement. ECG devices and cardiac monitors detect bioelectrical signals through electrodes attached to a subject’s body, while PCG recordings rely on acoustic signals captured via a microphone [39]. The PCG signal consists of two primary acoustic constituents: the systolic (S1) and diastolic (S2) heart sounds. The initial heart sound, S1, corresponds to the closure of the bicuspid and tricuspid valves at the onset of systole, aligning temporally with the peak of the R wave in the ECG signal. By contrast, the second heart sound, S2, originates from the closure of the semilunar valves, and its initiation and duration are linked to the T wave on the ECG signal [40].

Phonocardiograms also encompass additional heart sounds. The third heart sound, pre-diastolic (S3), is connected to valve muscle fluttering during rapid blood flow into the valves. Conversely, the fourth heart sound, presystolic (S4), signifies valve muscle fluttering during atrial systole. S3 and S4 are atypical in adults and indicate valvular insufficiency, recognized as the proto-diastolic and presystolic gallop [41].

**Figure 4:**
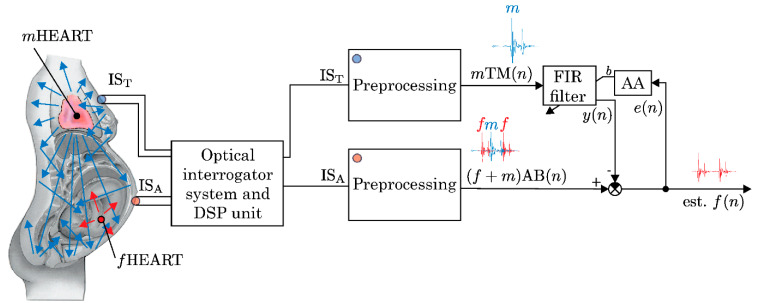
Basic schematic diagram of non-invasive PCG-based interferometric sensors and adaptive system for fHR monitoring. Reprinted from [38] under terms of the Creative Commons Attribution License.

In PCG, parameters including rate, frequency, duration, and alterations in specific segments of the captured cardiac acoustic signal are quantified, as illustrated in Figure 4. The integration of PCG and ECG signals empowers clinicians to diagnose a wide spectrum of heart conditions in both adults and fetuses [42,43]. The basic schematic diagram of a non-invasive PCG-based interferometric sensing system for monitoring fetal heart rate (fHR) is shown in Figure 4. Usually, the system consists of a coherent light beam emitting from a laser source. The mother’s belly is the target of this light, which interacts with the skin there. The reflected light beam is modulated by the mechanical vibrations of the fetal heart. A photodetector then records these modulations. The risk of harm is decreased by this non-invasive method, which enables the highly sensitive detection of fetal heart sounds without coming into direct contact with the fetal body. Furthermore, the interferometric technique can identify vibrations with extremely small amplitudes, which makes it especially helpful in situations where there is a low signal-to-noise ratio, like fetal heart monitoring.

Since PCG signals are acoustic in nature, outside noises like ambient vibrations, ambient sounds, and maternal body movements can interfere with them greatly. Accurately deriving information from the PCG signal can be challenging due to the potential for these external factors to mask the heart sounds. This sensitivity can result in low-quality signals and erroneous data, especially in non-clinical contexts where noise control is difficult [44,45]. Compared to adult heart sounds, the fetal heart’s auditory signals are comparatively feeble. Because of this, PCG systems need to be extremely sensitive in order to pick up on these weak signals, which can be challenging to obtain without increasing unwanted noise. Low signal-to-noise ratios caused by the weak fetal heart sound can make it more difficult to detect and analyze PCG signals with accuracy [46]. The location and movements of the fetus inside the uterus have a significant impact on the quality of PCG signals [47]. The resulting PCG signals may be weak or irregular if the fetus moves or is positioned in a way that reduces the heart’s accessibility to the sensors. Because of this reliance on fetal positioning, intermittent signal loss or the need to realign the sensors, which are both inconvenient and potentially disruptive to continuous monitoring, may result from this reliance.

PCG signals include possible murmurs or other acoustic events in addition to several heart sounds (such as S1, S2, S3, and S4). Particularly in a fetal context, where cardiac sounds are frequently more complex and less distinct than in adults, isolating particular sounds for analysis can be challenging. It can be difficult to correctly interpret the PCG data due to the complexity of the sound profile, especially when attempting to diagnose specific conditions based solely on these sounds [48]. Sophisticated signal processing techniques are needed to evaluate the timing and frequency characteristics of the heart sounds, filter out noise, and amplify weak signals in order to extract meaningful information from PCG signals. The complexity and cost of PCG-based monitoring systems may rise due to the requirement for sophisticated signal processing. Additionally, it requires much processing power, which is not always possible for portable or home-use devices. The correct placement of sensors has a major impact on PCG’s efficacy. Poor signal acquisition caused by improper sensor placement can reduce the system’s effectiveness or even render it useless. Due to its sensitivity to placement, setup may not always be feasible, particularly in at-home monitoring scenarios where users may not be technically proficient.

It can be difficult to conduct continuous long-term monitoring with PCG because stable sensor placement and steady signal quality over time are required. The sensor’s capacity to sustain excellent contact and signal quality can be impacted by variables like the movements of the mother, posture shifts, and skin conditions. These difficulties may make the use of PCG for continuous monitoring less practical, especially in home environments where conditions are less controlled than in medical facilities [49]. Due to variations in body type, skin thickness, and other anatomical factors that impact how sound waves travel through the body, PCG results can differ greatly between patients. Standardizing PCG measurements and interpretations may be challenging due to this inconsistency, which could lower the technique’s reliability across a range of patient populations. Although PCG is a well-researched method, little is known about its long-term efficacy in specific applications, like continuous fetal monitoring or the prediction of particular outcomes in high-risk pregnancies. The dearth of comprehensive, long-term research makes it difficult to fully comprehend the possible advantages and disadvantages of PCG in these situations, which could have an impact on clinical decision making.

### 2.4. Ballistocardiography (BCG) 

Ballistocardiography (BCG) is another reported noninvasive technique for measuring cardiovascular function. It is one of the few techniques for detecting mechanical motions caused by cardiovascular and cardiac activities like seismocardiography (SCG) [50], PCG, magnetocardiography (MCG) [51], and apexcardiography [52]. The basic principle behind BCG is rooted in the detection of the body’s micro-movements that occur with each heartbeat [53]. The heart exerts significant force when pumping blood into the major arteries, like the aorta, during each cardiac cycle. The entire body experiences a recoil force as a result of this blood ejection. The body experiences minor displacements or movements when blood is forcefully ejected from the ventricles into the artery system. These displacements are mostly vertical. The BCG waveform, which depicts the heart’s mechanical activity, is produced by processing the recorded movements. Waveforms generally exhibit discrete high points and low points that correspond to different stages of the cardiac cycle, including blood ejection, heart valve closure, and body recoil. BCG is measured through the reaction of the blood being pumped from the heart and can also measure blood volume and respiration, an activity that may be used to determine defects in the fetus [54]. By selecting the arterial blood pressure, any delays between the two primary sounds of the PCG may be found through BCG, and detecting both PCG with BCG can assist in a simplified blood pressure monitoring sensor [55]. While BCG can monitor heart rate and may become a reliable technique for detecting and monitoring fHR, the focus is currently on adults, amputees, sleep studies, and animal applications [56,57,58]. Table 2 shows a comparison between the discussed non-invasive fHR monitoring systems based on previous studies.

## 3. Commercially Available Fetal Monitoring Devices

Ultrasound is a method for diagnosing prenatal problems that typically employs a transducer that generates ultrasonic vibrations directed at the fetal heart or other organs. Ultrasonic waves reflect off the fetus’s structural components. Following the well-known Doppler Principle, they are detected by a suitable sensor and processed to determine the frequency shift caused by reflection from the moving fetal heart. Such information is then analyzed and integrated to offer fetal information, including heart rate. Hospitals and other public health facilities typically utilize this testing equipment. However, significant expenses restrict the duration of fetal monitoring, and high-accuracy ultrasound devices still need to be developed into a portable system that monitors fetal health as the woman undertakes her daily activities. Consequently, more than this, nonambulatory equipment is required for medical investigations attempting to assess the effects on the fetus of various environmental and behavioral factors and health patterns experienced or practiced by the mother. Furthermore, ultrasound procedures for detecting fHR are active, applying high-frequency ultrasonic vibrations to the growing fetus and fetal heart valve [59]. These invasive qualities may have a substantial negative impact on the fetus. Additionally, the alignment of the ultrasonic transducer with the fetal heart is relatively laborious. Even slight patient movement frequently leads to inaccurate readings and increased examination time and expenditures [59,60].

Indirect ST-segment analysis is provided by the STAN monitoring system, which is a commercially available monitoring device, using the waveform of the fetal scalp electrode. The STAN monitor made by Neoventa Medical in Gothenburg, Sweden, is an illustration of this [61,62]. In the late 1990s, European researchers integrated the STAN monitor into routine clinical practice. This approach hinges on analyzing the quantitative relationship between the amplitude of the fetal T-wave and R-wave, along with the presence or absence of a biphasic ST segment. This balance is employed to provide clinical reassurance or to raise concern when the fetal heart tracing exhibits characteristics that are neither ominous nor reassuring. This test draws on the observation that repolarization of the adult heart is highly sensitive to hypoxia, leading to ST segment elevation in individuals with coronary artery disease. The change in the amplitude ratio is believed to reflect altered cellular ionic currents occurring during anaerobic cardiac metabolism. Similar observations have been noted in fetal sheep, where experimental hypoxia induced elevation in ST-segment and T-waves in fetal ECG recordings.

Even in the absence of a typical ECG vector, it is possible to reliably compare the voltage amplitude of the T-wave and the R-wave, two ECG waveform components. This characteristic is important since fetal posture is frequently erratic and variable, making vector-based study of the fetal population difficult by nature [63]. This method of fetal cardiac monitoring has the intrinsic disadvantage of depending on the implantation of a fetal scalp electrode, which can only take place during labor. Additionally, only one dimension of the heart’s electrical activity can be seen with this electrode type. As a result, the technique’s sensitivity is severely limited by noise and is only useful for problems affecting the entire heart. These electrodes will not detect activities involving other cardiac areas or mild impacts. Fetal movement, which might appear as axis shifts, is another issue related to scalp electrodes. If several ECG leads are available, such problems could be addressed by providing more specific analyses earlier in pregnancy. Scalp electrodes are used in the STAN system [64], but an alternative to this invasive examination is a monitoring device with electrodes on the mother’s belly that can be administered at an earlier stage of pregnancy. 

**Figure 5:**
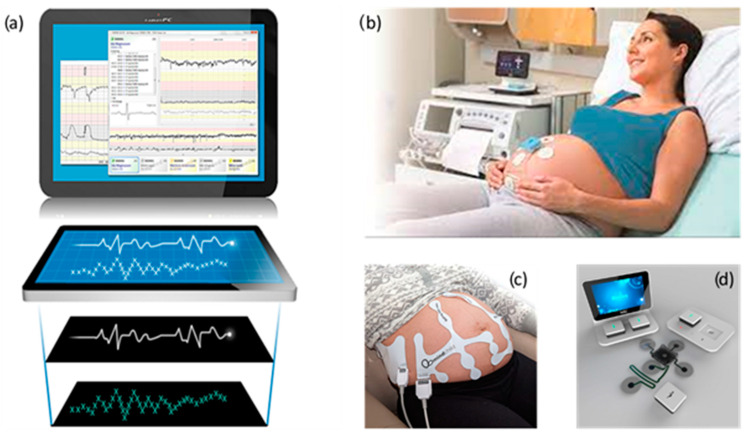
Different commercially available FHR monitoring systems (**a**) STAN monitors from Neoventa Medical, Goteborg, Sweden. (**b**) Monica AN24 Monitor from Monica Healthcare, Nottingham, UK. (**c**) MindChild Medical from North Andover, MA. (**d**) Novii, GE Healthcare, Cambridge, UK.

A transabdominal ECG is considered the leading commercial CTG monitor, as it offers a continuous transmission of signals and has the chance of being used when the mother moves during labor [65]. Commercially found monitors include the Novii Wireless Patch System (GE Healthcare, Buckinghamshire, UK), the next version of the Monica AN24 Monitor (Monica Healthcare, Nottingham, UK); MindChild Medical (North Andover, MA, USA); and Philips Avalon FM30 (Philips Healthcare, Amsterdam, The Netherlands), which uses triple-channel fetal monitoring; some variants are shown in Figure 5 [61,65,66]. The Monica AN24 TM (Monica Healthcare Ltd., Nottingham, UK) can obtain beat-to-beat fHR and fECG morphology signals with an effective sample rate of 2.1 kHz over extended periods. In a study, the Novii Wireless Patch system showed that it is more effective in people with BMIs over 30 kg/m^2^ and does help reduce mother and fetal HR confusion during the second stage of labor [66]. The Philips Avalon FM30 uses transabdominal ECG, Doppler, and FSE sensors, allowing mothers to move during labor [65,67].

Indications for continuous prenatal fetal electrocardiogram monitoring include high-risk pregnancies, such as fetal growth restriction (FGR), subsequent pregnancy after unexplained intrauterine mortality, and fetal cardiac arrhythmias [68,69]. Instead of an expensive hospital stay, the Novii Wireless Patch System can be coupled with a mobile phone application to enable fetal monitoring at home. The clinical feasibility of a fECG device relies on the detection of the fHR with precision, and these devices have demonstrated high accuracy scores of 88.5 ± 16.7% for ECG and 89.4 ± 7.6% for Doppler telemetry. The utility of the fHR signal hinges particularly on low rates of signal dropout to allow for reliable scoring of clinically significant fHRV measures. However, detecting the typically modest fECG from maternal abdominal surface electrodes is challenging due to interference from the maternal ECG. Due to maternal muscular activity, particularly from the abdomen, as well as other causes, it may be hampered [70]. Thus, it is crucial to delineate under which exact circumstances the fHR signal is available with fECG monitoring.

To accurately interpret fetal health, a representative and high-quality database is essential in evaluating any fECG analysis algorithm that may be developed for fetal heart monitoring devices [22,71,72]. Methods used to gather fetal ECG data can be classified as invasive or noninvasive. Invasive methods involve making direct contact between the recording electrodes and the skin of the fetus, a feat made only possible by intrauterine electrodes used during labor [73]. Although invasive procedures produce higher-quality signals than noninvasive ones, they are more inconvenient and can only be used to record during labor [74]. Conversely, noninvasive methodologies entail capturing signals from the maternal abdomen, a procedure feasible across all stages of pregnancy and involving the utilization of multiple electrodes [74]. Nevertheless, the method’s efficacy is curtailed by the low signal-to-noise ratio (SNR) of fECG and other concurrent interferences. Despite these limitations, owing to the manifold benefits inherent to the noninvasive approach, extensive research endeavors have been directed towards formulating signal processing strategies to extract fECG from noninvasively obtained recordings [75,76,77,78]. See Table 3.

## 4. Research in fHR Monitoring Systems

Currently, the primary focus of researchers engaged in this area is on fetal monitoring devices that automatically and continually check a fetus’s well-being. The ambulatory monitors in this area preferably have one or more fetal cardiac sensors for monitoring the fHR [79], and acoustic [7], electrocardiographic [73], or bioimpedance sensors [80] may also be utilized as required. These may also include interference sensors for monitoring the primary interference components of the fetal cardiac sensor signals. In monitoring fHRV, both the time domain analysis (TDA) and the frequency domain analysis (FDA) can be investigated [81]. These analyses are simple and offer high acceptance rates in clinical environments. These are captured by sensors and processed by software to be analyzed. 

### 4.1. Nonambulatory fHR Monitoring Patents

There have been several devices patented to analyze nonambulatory fHRs better. U.S. Pat. No. 2,536,527 designed a system for monitoring fetal condition during delivery with a microphone and a stethoscope to communicate and provide a signal that is amplified, filtered, rectified, and utilized to activate two relays that indicate unusually high or low fetal heart rates or amplitudes [80]. U.S. Patent Number 3,187,098 unveils a fetal heartbeat detection apparatus that employs the intrinsic frequency of the Farrar detector. This apparatus integrates a cantilevered piezoelectric crystal positioned within a contacting slab, operating at approximately 50 Hz [82]. Another fetal monitoring device is presented in U.S. Patent Number 3,409,737, featuring the Settler monitor. This monitor is worn with a microphone belt incorporating three microphones. The system includes a three-stage amplification circuit that enhances the fetal heartbeat signal while simultaneously eliminating maternal heartbeat interference [83].

U.S. Patent Number 3,599,628, attributed to Abbenante et al., introduces an apparatus for monitoring fetal heartbeat and intrauterine pressure. This device aims to establish a correlation between these two parameters, thereby indicating fetal distress. In this system, intrauterine pressure is assessed by inserting a catheter directly into the uterus, which is connected to the fetus. This catheter is coupled with a piezoelectric pressure transducer to facilitate the measurement of fetal electrocardiography (ECG) during the delivery process [84]. U.S. Patent Number 3,703,168 reveals a fetal heart monitoring system equipped with electrodes placed on the mother’s skin to extract an electrical signal from that location. This signal captures both fetal electrocardiography (ECG) and an indicator of maternal contractions derived from the electrode signal [85]. To measure the decelerations in fHR during uterine contractions, Sureau et al. unveil an fHR device that can be used during labor [86]. U.S. Patent Number 4,299,234, authored by Epstein et al., presents an fHR (fetal heart rate) monitor that merges ECG and EMG signals to enhance the dependability and precision of the resultant heart rate data [87].

### 4.2. Ambulatory fHR Monitoring Systems

Due to their traditional solitary function of analyzing an input signal to estimate fHR, prior fetal monitors have been limited in their utility. Some have also utilized intrauterine pressure as an additional indicator of fetal stress. However, none were intended to provide an ambulatory monitor capable of continually and automatically analyzing fetal health using more complicated analysis based on the fetus’s heart rate patterns and movements. Due to the lack of such an ambulatory fetal monitor in the prior art, it has been hard to properly monitor and notify a mother of possible fetal health hazards, such as those resulting from maternal activities. 

Wearable technology, which can offer continuous and simultaneous monitoring of fHR and movement outside the clinic, could detect the onset of fetal deterioration and permit intervention before fetal death [60]. This surveillance would benefit women with high stillbirth risks and low-risk pregnancies to prevent the occurrence of stillbirths without evident complicating causes. Yuan et al. created a portable, low-power fetal ECG collector that gathered a mother’s abdominal ECG signals in real-time. The ECG data were transmitted over Bluetooth to a smartphone client, and based on the Android operating system, a mobile application software was developed for this system. The application integrated the “fast fixed-point algorithm” for independent component analysis (FastICA) along with the “sample entropy technique” to achieve real-time extraction of fetal electrocardiogram (ECG) signals from maternal abdominal ECG signals. Using retrieved fetal ECG signals, the fHR was calculated. The experimental results demonstrate that the sample entropy may accurately determine the channel where the fetal ECG is located. However, the algorithmically recovered ST segment of the fetal ECG signal was severely contaminated by noise. The suggested approach was evaluated using maternal abdominal ECG data simulated by a maternal abdominal signal generator [22].

**Figure 6:**
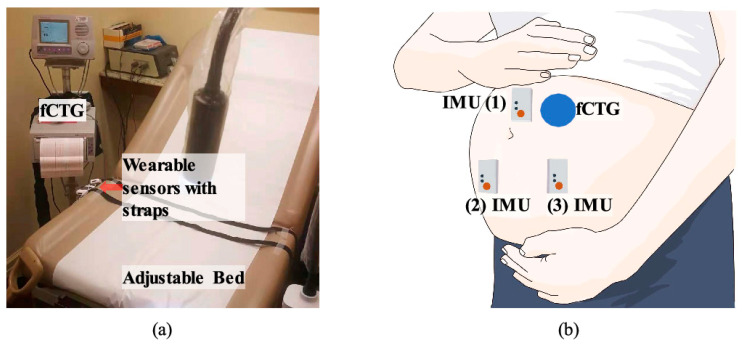
(**a**) Experimental setup and environment. (**b**) Illustration showing the proof-of-concept setup with three sensor nodes Reprinted from [88] with permission, Copyright © 2024 Elsevier B.V.

Preparing a proof-of-concept configuration using commercially available sensor nodes, Yang et al. proposed a novel approach for detecting fHR using seismo-cardiogram (SCG) and gyro-cardiogram (GCG) recordings collected from abdominal inertial sensors (Figure 6). The viability of the suggested method was examined using data collected from 10 pregnant women in supine, seated, and standing postures. The fHR components are taken from the combined cepstrum of all sensor records, and the results were compared with concurrently obtained fetal CTG recordings (fCTG). This investigation determined that the optimal posture for signal collection is the supine position, with an average root mean square error (RMSE) of 9.83 BPM and a moderately favorable percentage of agreement (PPA) for the SCG signal. The total RMSE readings for SCG are 11.40 BPM, and those for GCG are 12.08 BPM. SCG’s absolute reliability is 75.02%, somewhat lower than GCG’s 75.52%. In summary, the results are comparable between the two modalities, suggesting no significant difference between the usage of the two methods. The results imply that wearable inertial sensors may be used outside of clinical settings to precisely and consistently extract fHR, attaining precision and reliability parameters that are comparable to those of other modalities like fCTG. Eliminating motion artefacts caused by maternal and fetal movement is a crucial obstacle for the suggested technology [88].

Hu et al. proposed a flexible, wearable, wireless fetal ECG (ECG) monitoring system. A multiunit, integrated electrode, transmission line, and processing circuit structure was built using micromachining and laminated packaging technology. Serpentine wire was used to achieve the device’s pliability with five flexible electrode patches. These were utilized to create numerous measurement and interference elimination channels; the singular value decomposition technique was integrated to improve measurement accuracy. The signal acquisition device weighs approximately 12.5 g, and the electrode patch is only 0.25 mm thick. This device’s ability to completely adapt to the skin substantially improves its wearability. Data can be communicated wirelessly via Bluetooth through this device, enabling measurement portability at any time and place. Clinical application results demonstrate that the system accurately detects fHR, the mother’s heart rate, and uterine contractions with results in good accord with the commercial Philips fetal monitor. It is reported that this technology can measure fHR continuously for more than six hours, and it provides robust assurance for fetal health monitoring [89].

Sarafan et al. developed a novel wearable fetal electrocardiogram (fECG) monitoring system consisting of an abdominal patch communicating with a smart device. The system’s primary components are the fetal patch and the monitoring app. The fetus patch’s electronics and recording electrodes were constructed on a hybrid flexible–rigid chassis, whilst an Android application was designed for various uses. The patch collects abdominal electrocardiogram (aECG) signals, which are then transmitted to a mobile app using secure Bluetooth Low Energy (BLE) transmission. The app software connects to a cloud server where processing and extraction algorithms are conducted to calculate the fHR and extract the fECG from raw data in real time. They reported success with the algorithms and real-time data recorded on pregnant subjects, yielding promising outcomes. The method promises to adapt the currently utilized fetal monitoring system into effective telematernity care over long distances [90].

Zhang et al. proposed a portable ECG monitoring system to record the pregnant woman’s abdominal ECG (aECG), comprising both maternal ECG and fECG, which could be applied to fHR monitoring in a home setting. By integrating data acquisition circuits, a data transmission module, and a signal processing platform featuring low input-referred noise and high input impedance, a high-resolution ECG monitoring system was devised. To validate its efficacy, abdominal electrocardiograms (aECGs) were recorded and analyzed from pregnant women assuming three distinct positions (supine, seated, and standing). The results illustrate that the proposed device can reliably record aECGs in diverse postures, maintaining signal quality and precision for both fetal ECG (fECG) and heart rate data. The performance of fetal QRS (fQRS) complex extraction is evaluated using sensitivity (Se), positive predictive accuracy (PPV), accuracy (ACC), and their harmonic mean (F1). Notably, the average Se, PPV, ACC, and F1 scores for fQRS complex extraction stand at 99.62%, 97.90%, 97.40%, and 98.66%, respectively. This research demonstrates the promising application of the suggested method in fetal health monitoring. However, only short-term recordings in three postures (supine, seated, and standing) have been tried, and extended monitoring periods with consistent signal quality have yet to be experimentally shown. In addition, the investigation used only a few subjects [30].

Braun et al. developed a wearable system based on CSEM’s cooperative sensors. This adaptable technology enables the measurement of several biosignals and the simple incorporation of a patch or garment. Twenty-five patients with singleton pregnancies and gestational ages less than 37 weeks were used to test the method. To reject inaccurate fHR calculations, the method for signal processing gives a signal quality index. A commendable performance in fetal heart rate (fHR) estimations was achieved among 12 out of the 21 participating patients, yielding a mean absolute error of five beats per minute (bpm) and an acceptance rate exceeding 70%. However, the remaining nine patients had poor acceptance and significant error rates, and the source of these high error rates was investigated [91]. 

## 5. Textile-Based Fetal Heart Monitoring Systems

The growing interest in wearable electronics leads to textile-based devices that continuously monitor physiological signals and provide the comfortability and portability of clothing [92,93]. Textile-based wearable sensing devices have been used in various applications, such as for rigorously monitoring athletic performance and clinical and health monitoring [94]. At this time, different bio-potential sensing wearable textile devices for monitoring ECG, EMG, and EEG exist [95], such as textile-based wearable systems for the unobtrusive recording of cardio-respiratory signals and using t-shirts with electrodes [96,97]. For non-invasive fetal monitoring systems, textile-based sensors provide a considerable advantage over traditional devices because of their flexibility, comfort, washability, ability to work without gel, quality of being less cumbersome than some current systems, and opportunity to be used at home [15,39,98,99]. Apart from their intrinsic benefits, the effectiveness of textile-based fetal heart monitoring systems can be assessed through multiple crucial parameters. It is crucial that textile-based sensors are able to deliver precise and high-quality fetal heart rate (fHR) signals. To guarantee accurate readings even in the face of movement or outside interference, this involves preserving a high signal-to-noise ratio (SNR). In order to correctly differentiate weak fetal heart signals from maternal heart signals and other physiological noises, the sensors must have sufficient sensitivity. The comfort of the wearer is crucial because these devices are meant to be used continuously, particularly in high-risk pregnancies. Breathability, non-irritability, and sufficient comfort for extended wear are the qualities that the materials should have. The performance of textile-based sensors must not deteriorate even after frequent washing and wear. This includes preserving sensor integrity and electrode conductivity following numerous washing cycles. To enable widespread adoption, textile-based sensor fabrication should be scalable and affordable. One example of this is the capacity to incorporate sensors into different kinds of fabrics and clothing without appreciably raising the cost of manufacturing.

### 5.1. Fabric Structures in Textile-Based Fetal Monitoring Systems

The performance of wearable sensors is significantly influenced by the structural design of textiles. The fabric selection influences the device’s flexibility and comfort, as well as the stability of the sensors, which is essential for obtaining reliable readings over time.

#### 5.1.1. Woven Fabrics

To create a stiff structure, two sets of yarns are interlaced at right angles to create woven fabrics. Although woven fabrics have higher dimensional stability and durability, their rigidity can make them less appropriate for applications that need flexibility, like wearable fetal monitoring. Nonetheless, they can offer a reliable foundation for embedding sensors in situations where accurate sensor placement is essential. In certain instances, woven materials are combined with more flexible fabrics to balance comfort and durability in wearable systems. Woven structures in fetal monitoring could be taken into consideration for applications where consistency in sensor placement and long-term durability are of utmost importance.

#### 5.1.2. Knitted Fabrics

The structure of knitted textiles is extremely flexible and stretchable because of the interlacing of yarns. Since knitted fabrics must adapt to the body’s changing shape during pregnancy, their elasticity makes them perfect for wearable technology, particularly in fetal monitoring. Knitted textiles offer constant skin contact to reduce signal loss and preserve sensor performance even when moving. According to studies, conductive yarns like silver-coated fibers can be incorporated into knitted fabrics to detect fHR signals effectively while allowing for a comfortable fit for the wearer.

#### 5.1.3. Non-Woven Fabrics

Unlike woven or knitted fabrics, non-woven materials are made of fibers fused by mechanical, chemical, or thermal means. These materials have low durability but are lightweight, breathable, and frequently used in disposable applications. Consideration is being given to non-woven fabrics for short-term or one-time use monitoring systems, especially in clinical environments where cost and hygienic conditions are essential factors. Short-term fetal monitoring applications may benefit from non-woven fabrics despite their lower durability than woven or knitted alternatives.

### 5.2. Sensor Materials in Textile-Based Fetal Monitoring

The electrical characteristics, biocompatibility, and general performance of a sensor are all greatly influenced by the material selection. Flexible wearable sensors that can identify weak electrical signals, like those from the fetal heart, are frequently made using conductive fibers and polymers.

#### 5.2.1. Metal-Coated Fibers (Silver, Stainless Steel)

Excellent electrical conductivity and biocompatibility make silver-coated fibers a popular choice for textile-based sensors. Since their high conductivity enables accurate detection of fHR signals, spandex blends or silver-plated nylon are commonly used in fetal monitoring systems. These fibers impart flexibility and comfort without compromising performance when woven or knitted into textiles. Although less conductive than silver, stainless steel fibers are more durable and frequently combined with other materials. Stainless steel is prized in fetal monitoring systems for its ability to withstand corrosion, even though it typically shows greater resistance, which may have an impact on the sensor’s sensitivity. In an attempt to produce knitted electrodes, researchers have experimented with combining polyester and stainless steel; however, the results show that these materials do not capture high-quality fHR signals as well as their silver-coated counterparts.

#### 5.2.2. Conductive Polymers

Conductive polymers like PEDOT, PANI, and polypyrrole (PPy) have drawn interest because of their adaptability, low weight, and simplicity of incorporation into textiles. By doping or combining with metal nanoparticles, these polymers can increase their moderate electrical conductivity. Conductive polymers provide a cost-effective substitute for metal-based fibers in fetal monitoring, especially in situations where comfort and flexibility are essential. Nonetheless, there are still issues with these polymers’ long-term resilience and conductivity stability, particularly after repeated exposure to washing cycles.

#### 5.2.3. Hybrid Materials

Hybrid materials that combine metal nanoparticles or polymers with conductive fibers to enhance performance have been the subject of recent research. For instance, it has been demonstrated that high conductivity can be achieved while retaining the flexibility needed for wearable applications when silver nanoparticles are embedded in cotton or nylon fibers. The performance, comfort, and cost-effectiveness of hybrid materials make them a desirable choice for creating scalable fetal monitoring systems.

### 5.3. Fabrication Methods for Textile-Based Fetal Monitoring Sensors

#### 5.3.1. Embroidery and Sewing

Functional sensors are made through embroidery techniques, which entail sewing conductive threads or fibers directly onto textiles. This technique can precisely position sensors to maintain stable skin contact and increase signal accuracy. Additionally, embroidered sensors are very resilient and can withstand numerous washing cycles without experiencing a noticeable reduction in conductivity. Studies have shown that the performance of embroidered sensors used in belts or clothing to provide continuous fHR monitoring is comparable to that of conventional gel electrodes in fetal monitoring applications.

#### 5.3.2. Screen and Inkjet Printing

Printing technologies, including inkjet and screen printing, are scalable for integrating sensors into textiles. To create thin, flexible sensors, conductive inks, usually containing carbon or silver nanoparticles, are printed onto fabric substrates. These techniques make it possible to control sensors’ placement and design precisely, making them ideal for producing small, discrete devices. Printed sensors are ideal for long-term use because of their flexibility and ease of integration into wearable clothing. They have been successfully used in various applications, including fetal monitoring.

#### 5.3.3. Weaving and Knitting Conductive Fibers

Another technique for creating seamless, integrated sensors is to directly weave or knit conductive fibers into fabrics. Since the entire fabric structure functions as a sensor, knitted conductive sensors in particular provide a high level of comfort and flexibility. With the added advantage of being washable and reusable, knitted silver-coated fibers have shown excellent accuracy in fetal heart rate signal detection. An even more stable structure can be achieved by weaving conductive fibers into fabrics; however, this method is less flexible than knitting, so it is not as good for applications that require frequent movement.

Maternal and fetal monitoring should ideally be undertaken continuously, day and night while undertaking daily activities. Therefore, monitoring the fHR from home is beneficial, especially with difficult or high-risk pregnancies. The mother can use these systems, and they contain wireless technology that can send the data collected to the doctor without requiring in-office visits [100,101]. Table 4 shows different proposed textile-based, dry electrodes for fHR monitoring, their fabrication methods, and if they can be compared to standard gel electrodes.

### 5.4. Recent Progress in Textile-Based Fetal Heart Monitoring Devices

**Figure 7:**
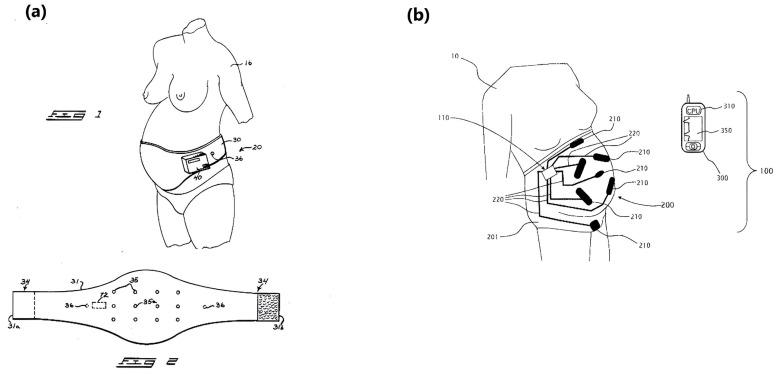
Schematics of (**a**) U.S. Patent US4781200A and (**b**) US Patent US20170150926A1. Reproduced from [106,107] under the Creative Commons Attribution License.

Fetal monitoring systems are routinely placed around the mother’s abdomen through electrodes attached to the skin or a band that fits snugly against the body with electrodes placed underneath. Textile-based monitoring systems change this by fabricating the electrodes directly into the textile so that it is one device instead of connecting numerous parts. Donald A. Baker patented a self-contained, lightweight ambulatory fetal monitoring system capable of conducting a substantially continuous analysis of fetal well-being, as seen in Figure 7a [106]. The monitor is a sensor-equipped garment incorporating several sensors a pregnant mother can wear. The device features an alarm system for fetal behaviour deviating from the preprogrammed parameters, an acoustic sensor for fHR detection, ECG sensors, and a wireless processing unit connected to a watch. This device allows at-home monitoring and is a simple belt that a mother can quickly wear without extra cords/devices needing to be placed on her abdomen. 

U. Amir created a patent that utilises a knitted or woven garment with various conductive textile electrodes for detecting maternal and fetal electrical vital signs, as shown in Figure 7b [107]. This clothing facilitates the integration of multiple electrodes, enabling the monitoring of various parameters such as oxygen saturation, respiration rate, skin temperature, blood pressure, ECG parameters (including ST elevation and depression), maternal body posture, and movement. Additionally, it encompasses the tracking of fetal heart rate (fHR), uterine contractions, and electromyography (EMG) activities. A specific yarn is incorporated into the clothing and connects each electrode to a processor, which can be snapped onto the garment. These processors communicate the data wirelessly to a specified device. This wireless feature allows at-home monitoring while allowing the doctor to give supervision and guidance. 

Another patented device uses a piezo film sheet with two electrodes to receive biopotential signals [98]. The device detects the maternal heart rate, uterine activity, fHR, and fetal motion using PCG and ECG technologies. The PCG detects a strong fetal acoustic signal and fetal motion. Breathing and maternal/fetal motion (movement) artefacts are cancelled to improve the quality of the uterine activity detection from the uterine EMG signals found underlying the abdominal ECG signal. In this system, no skin preparation is needed, which is advantageous, as no gel needs to be applied or cleaned. Also, this system covers a substantial portion of the abdomen so that the SNR and the PCG signal can capture the fHR in multiple directions. Because the PCG is independent of the fetal position in the uterus, there is a reduction in signal loss in fHR determination, which can assist in fetal heart monitoring in obese patients [98]. Finally, optimising the at-home application of this device, a passive technology is used, incorporating sleep or low power modes when a maternal pulse is present, therefore minimizing battery use and maximising battery life.

Manna et al. proposed a wearable fetal ECG monitoring system for high-risk pregnant women, which can be a solution for a personal health monitoring system in developing nations to lower the birth-related death rate [102]. The acquired data can be communicated to an expert or hospital, and opinions can be received without regular clinic visits. A customised pattern of sensors embedded into a fabric is used to acquire the fetus’ and mother’s physiological signals. The physiological signals are analysed to determine the fetal and maternal physiological status. The result demonstrates excellent concordance with the conventional fetal ECG recording. 

Manna and R. continued this research by fabricating an fHR monitoring system from dry electrodes made from conductive fibers. The conductive fibers chosen were a blend of silver nanoparticles/nylon yarn and a mix of silver/cotton/spandex due to their electrical stability when exposed to sweat, antibacterial properties, and anti-allergic properties [103]. Ten electrodes were sewn onto a belt; eight were placed on the mother’s abdomen and two on the mother’s chest to measure the fECG. The silver and nylon sensors had a meager resistance of less than one ohm per foot, allowing the device to acquire fECG signals equal to those obtained by a gel electrode. This device requires more research to lower the SNR and develop a wireless system but shows how textile-based electrodes can be applied to fHR monitoring. 

Another textile-based wearable diagnostic or analytical subsystem within the signal processing system is designed to conduct a diagnostic test for fetal health, also known as the fetal non-stress test [108]. In essence, the non-stress test involves the identification of fetal movement and the continuous monitoring of fHR to determine whether a satisfactory increase or acceleration has transpired. The inability of the fetus to react normally may suggest fetal distress caused by various factors. Medical interpretations of the specific numbers used as test parameters are prone to variation, and one set of parameters needs a second fHR acceleration cycle, including at least beats per minute over a recorded baseline fHR [109,110].

**Figure 8:**
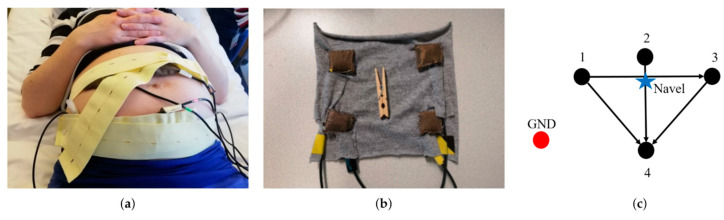
A textile-based sensor. (**a**) Placement of electrodes using elastic belts on the mother’s belly. (**b**) Four textile electrodes. (**c**) Schematic configuration of the electrodes and the related bipolar leads, which are identified by arrows. The design is symmetric concerning the navel. Reproduced from [99] under terms of the Creative Commons Attribution License.

A proficient fECG was constructed by using conductive fabric that does not require gel, allowing increased impedance and, therefore, lower signal amplitude, as shown in Figure 8 [99]. This device is made from four textile electrodes placed on the fabric and placed on the mother’s abdomen, allowing for a comfortable, unobtrusive process of acquiring the needed signals. The electrodes had high resistance and resulted in charge accumulation and artefacts, which need to be addressed in future research to increase the system’s robustness. Researchers currently use an elastic band to hold the fabric electrodes in place. Nevertheless, once the optimum electrode configuration is found, the electrodes can be fabricated onto a garment. 

**Figure 9:**
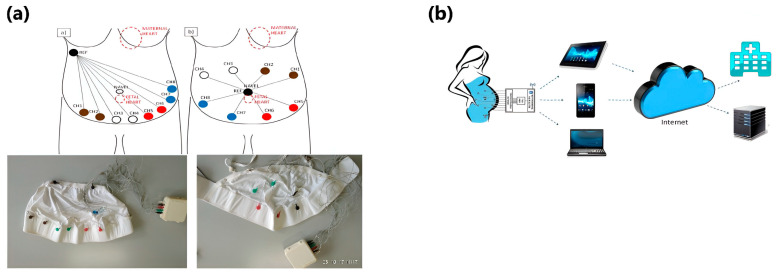
Stretchable fHR monitoring device. (**a**) Schematic of the relative position of the electrodes on the mother’s abdomen and the fabrication of the sensors on the bodysuit; (**b**) a functional diagram of the Telefetalcare System. Reproduced from [104] under terms of the Creative Commons Attribution License.

Using cotton and stretchable Lycra^TM^, a bodysuit for pregnant women, allows the woman to wear the garment without worrying about sensor placement or if the garment fits correctly [104]. This research follows research conducted by two groups who worked on a bodysuit with silver yarn electrodes and the telemonitoring service [111,112]. As shown in Figure 9, the garment envelops the pregnant body and holds the silver yarn electrodes in place. The electrodes are sewn directly onto the Lycra^TM^, allowing for close contact with the abdominal skin, and no gel is needed. Bluetooth carries the data to a smartphone, allowing at-home care, better examination performance, and more personalised care. This device has an overall accuracy and sensitivity score of over 90%. 

Using conductive yarn to make the electrode is a popular method for fabricating textile-based fHR monitoring systems. Another research group used stainless steel with polyester fibers added at specific percentages to make a knitted sensor [105]. Six electrodes were placed on a belt that would wrap around the pregnant woman’s abdomen and measure the fHR. Stainless steel was used for its low resistance, but only the 100% stainless steel electrode could record the fECG. The 100% stainless steel sensor weighed 2.9 g/m^2^, had a thickness of 1.77 mm, and was not knittable, so it was sewn instead.

## 6. Conclusions

Heart rate variability in response to uterine activity as measured by CTG is one of the approaches used for evaluating fetal health and has a relatively low specificity despite its excellent sensitivity. In obstetrical practice, the heart rate is typically detected by placing a non-invasive Doppler ultrasonography probe on the mother’s abdomen or an invasive electrode on the fetal scalp. The first method is relatively imprecise yet is applied throughout the pregnancy. The latter approach is significantly more precise, but it can only be used after the rupture of the membranes and sufficient dilation, limiting its application up to the final stages of pregnancy. As a supplementary measure to CTG, incorporating fECG monitoring might enhance the precision of fetal distress detection. However, the challenge lies in the low SNR encountered when attempting to record fECG from the maternal abdomen non-invasively. Furthermore, the positioning of the fetus within the mother’s uterus can introduce variability in the fECG tracings observed on the abdomen. 

The integration of smart textile devices for fHR monitoring has the potential to revolutionise maternal healthcare monitoring. This is attributed to the capability of smart textile-based electrodes to significantly enhance patient comfort while alleviating skin discomfort, particularly during extended monitoring periods. In recent years, there has been an increase in interest in smart textiles as tools for continuous long-term monitoring of physiological signals, such as ECG, due to their superior comfort and portability compared to conventional monitoring devices. However, using smart textile electrodes in fHR monitoring is still in the beginning stages of research. It requires more investigation into the fabrication method of the monitoring system, the number of electrodes needed, and improving the signal quality of fHR due to its smaller amplitude compared to the mother’s heart rate.

## 7. Future Perspectives

One of the most innovative areas of prenatal care is the future of fHR monitoring, especially with the development of textile-based wearables. The non-invasive, continuous, and more individualised monitoring capabilities of these rapidly developing devices promise to completely change the landscape of fetal monitoring. In addition to strengthening these devices’ technical capabilities (accuracy and dependability), the next stage of research and development should put equal emphasis on aspects that are important to users, like comfort and simplicity of use.

A major opportunity for predictive analytics in fetal health is presented by the combination of artificial intelligence (AI) and machine learning (ML). Artificial intelligence (AI) in prenatal care may provide a new avenue by enabling earlier and more precise identification of possible problems. But to properly integrate technology, it is important to consider the privacy and ethical issues surrounding AI and data handling in the healthcare industry.

Cooperation will be essential in this rapidly changing field. Interdisciplinary teams comprising engineers, healthcare professionals, and data scientists are indispensable to ensure that these cutting-edge technologies are technically sound, clinically relevant, and ethically sound. These collaborations can navigate the regulatory environment, which is essential for the clinical adoption of these technologies.

Future research should consider these technologies’ global implications. One of the biggest challenges is increasing access to high-quality prenatal care, particularly in rural and impoverished areas. With the ability to monitor remotely, textile-based wearables can potentially be a major solution to this problem, improving the accessibility and equity of prenatal care.

The development of continuous, non-invasive fetal heart monitoring through textile-based monitoring systems has demonstrated great promise. These devices are the best for long-term maternal and fetal health monitoring because of their unmatched comfort, portability, and ability to blend in with regular clothes. Nevertheless, several obstacles must be overcome to fully utilise these systems’ potential in home and clinical settings. Ensuring signal acquisition is accurate and reliable is one of the main problems with textile-based monitoring systems. Future research should concentrate on creating sophisticated materials and electrode designs to improve contact quality and lessen motion artefacts. This includes developing more advanced signal processing algorithms to reduce noise, boosting the signal-to-noise ratio (SNR), and investigating innovative textile materials that can sustain constant signal quality even when moving.

Enhancing the sensors’ sensitivity and specificity will help them distinguish weak fetal heart signals from other physiological signals like a mother’s heartbeat and muscle contractions. This could entail creating multi-sensor systems that can triangulate signals to give a more precise reading or applying machine learning methods to differentiate between various signal kinds. These systems produce enormous volumes of data that must be processed and sent in real time since they are meant to be used continuously. To efficiently handle large amounts of data, future research should investigate methods to optimise data processing algorithms. One way to lessen the strain on central processing systems is by developing edge computing solutions, which can process data locally on the device before transmission.

Furthermore, studies should concentrate on enhancing wireless data transfer methods to guarantee dependable and safe communication between wearable gadgets and medical professionals. One way to ensure this is to investigate high-bandwidth, low-power wireless protocols that can handle constant data streaming without rapidly depleting the device’s battery. Future studies should improve the scalability and affordability of the textile-based sensor fabrication process to make these systems broadly accessible. This entails creating new manufacturing processes that generate affordable, high-quality sensors, allowing for broader adoption in developed and developing nations.

Integrating textile-based monitoring systems with other wearable or in-home monitoring devices to create a more complete ecosystem for monitoring maternal and fetal health is another exciting avenue for future research. This might entail integrating blood pressure, respiratory, and other vital sign monitoring with heart rate monitoring to provide a comprehensive picture of the health of the mother and fetus. Successful implementation of these textile-based systems depends on ensuring their long-term usability and durability. Future studies should examine how long these sensors can be worn, including whether they can be repeatedly washed and worn without losing functionality.

## Figures and Tables

**Table 1:** Fetal and maternal heart rate data.

	Heart Rate (HR) BPM	Expected HR BPM	QRS Spectral Energy (Hz)	Peak-to-Peak Amplitude (μV)
**Fetal**	60–240	140	20–60	3–25
**Maternal**	50–210	80	10–30	100

**Table 2:** Comparison between FHR monitoring techniques.

Method	Advantages	Limitations	Gestational Age	Energy type	Accuracy Issues in FHR Monitoring	Installation Cost
fECG	Cost-effective, user-friendly, continuous long-term monitoring, enabling precise beat-to-beat variability tracking for comprehensive fetal heart assessment	Complex design requirements, dependency on fetal orientation in utero, signal quality variations due to maternal and fetal movement, potentially leading to data inaccuracies	Around the 20th week of gestation onwards	Electrical	Accuracy can be compromised by signal interference from maternal ECG and movement artifacts, leading to potential false readings.	Moderate to high, depending on the complexity of the system and the need for specialized electrodes
fCTG	Economic, low-power, safe, and versatile monitoring capabilities, making it easily manageable, suitable for extended recordings, adaptable to clinical environments, portable, and viable within MRI settings	May result in a higher rate of false positives and interventions due to its sensitivity to maternal factors and reduced specificity in predicting fetal distress	Around the 28th week onwards	Ultrasound	Potential for false positives due to sensitivity to external factors, leading to unnecessary interventions	Low to moderate, typically involves standard ultrasound equipment
fBCG	Non-invasive and continuous fetal heart activity monitoring provides a low-cost, radiation-free, and sensitive method for long-term pregnancy assessment	Accuracy can be affected by factors like fetal position and movement, potentially leading to inconsistent readings	Usually from the third trimester	Mechanical	Inconsistent readings due to fetal movements and sensitivity to maternal positioning, which can affect accuracy	Low to moderate, generally less expensive due to fewer required components
fPCG	Non-invasive and safe means of monitoring fetal heart sounds, facilitating early detection of anomalies and providing valuable insights into fetal well-being during pregnancy	Monitoring might be prone to external noise interference, which could impact the accuracy of detecting fetal heart sounds	Around the 20th week of gestation	Acoustic	Susceptible to external noise and maternal movement, which can cause inaccuracies in detecting fetal heart sounds	Low, as it primarily requires a stethoscope or microphone system

**Table 3:** The advantages and disadvantages of the fetal monitoring technologies that are currently on the market.

Device/Method	Advantages	Disadvantages	Applications/Use Case	Specific Devices
Ultrasound	Widely used in hospitals and public health facilities. Offers detailed fetal information, including heart rate. Portable versions for continuous monitoring still under development.	High cost limits the duration of monitoring. Non-ambulatory; requires clinical setting. Laborious alignment of transducer with fetal heart. Inaccurate readings due to patient movement.	Commonly used for prenatal diagnostics to monitor fetal heart rate and other organs. Not suitable for continuous, long-term monitoring. Limited by high-frequency ultrasound vibrations. High cost and specialized training required.	Standard ultrasound equipment.
STAN Monitoring System	Provides ST-segment analysis using fetal scalp electrodes. Analyzes the relationship between T-wave and R-wave for clinical assessment.	Requires invasive procedure (fetal scalp electrode). Limited to the labor stage; not applicable earlier in pregnancy. Sensitivity limited by noise; difficult to detect activities affecting specific areas of the heart.	Used during labor to monitor fetal heart health. Particularly useful for high-risk pregnancies.	STAN Monitor (Neoventa Medical)
Transabdominal ECG (fECG)	Continuous signal transmission, even with maternal movement. Non-invasive; allows for monitoring at home when coupled with a mobile application. High accuracy for ECG and Doppler telemetry (88.5–89.4%).	Signal quality can be affected by maternal ECG and abdominal muscle activity. Low SNR due to interference from maternal ECG. Challenging to extract clear signals due to noise and interference.	High-risk pregnancies (e.g., fetal growth restriction, arrhythmias). Can be used from earlier stages of pregnancy up to labor.	Novii Wireless Patch System (GE Healthcare) Monica AN24 Monitor (Monica Healthcare) Philips Avalon FM30 (Philips Healthcare)
Fetal Phonocardiography (fPCG)	Non-invasive and safe; detects fetal heart sounds.	Highly susceptible to external noise interference.	Early detection of cardiac anomalies.	Standard phonocardiography devices.
MindChild Medical	Simple and low-cost setup. Continuous fetal monitoring with advanced technology. Portable and adaptable to different environments.	Limited by the acoustic properties of the maternal abdomen. Installation and operational costs might be high. May require specialized training for effective use.	Can be used from the 20th week of gestation. Used in clinical settings for detailed fetal monitoring. Suitable for long-term and high-risk pregnancy monitoring.	MindChild Medical Monitor

**Table 4:** Fabricated dry electrodes for fHR monitoring.

Chosen Yarn	Fabrication Method	Comparable to Gel fECG?
Silver Plated Nylon [102]	Electrodes sewn onto belt	Yes, but uncertain which electrode worked
Cotton/Silver/Spandex [102]	Electrodes sewn onto the belt	Yes, but uncertain which electrode worked
Silver Plated Nylon [103]	Electrodes sewn onto the belt	Yes, but uncertain which electrode worked
Cotton/Silver/Spandex [103]	Electrodes sewn onto the belt	Yes, but uncertain which electrode worked
40% Stainless Steel/60% Polyester [99]	Knitted electrode that is sewn onto the belt	No
80% Stainless Steel/20% Polyester [99]	Knitted electrode that is sewn onto the belt	No
100% Stainless Steel [99]	Sewn electrode that is sewn onto the belt	Yes
Conductive Fabric [104]	Held in place by an elastic belt	Yes
Silver Yarn [105]	Yarn sewn directly on cotton/lycra bodysuit	Yes
